# Progress in Gynæcology

**Published:** 1903-03-21

**Authors:** 


### Progress in Gynæcology.

(*Concluded from page* 410.)

**Retroflexion of the Uterus.**—W. J. Gow,[8] in a paper on "Uterine Displacements," draws attention to the important part that the muscular tone of the uterus plays in maintaining that organ in its normal position. There is no doubt that in certain people the uterus is not always in the same position. A uterus which, on first examination may be found to be directed backwards, may a week later, or possibly even a day later, be found in the more ordinary attitude of anteversion and flexion, and this without any form of treatment, the alteration in position depending to a great extent on the muscular tone of the viscus. He points out that either retroflexion or marked anteflexion may occur as a congenital condition, and that the dysmenorrhœa and other symptoms complained of in these cases are due, not so much to the displacement as to the imperfect development of the organ. With regard to treatment, he thinks that in many cases the symptoms can be relieved by the use of a pessary, the watch-spring and Hodge being the most suitable. He insists that retroflexion is a condition which is perfectly unalterable by any pessary introduced into the vagina, and that the insertion of a pessary in these cases relieves the symptoms, not by making the body of the uterus look forward, but by curing the prolapse which is always present. In cases where the symptoms are unrelieved by a pessary, or the patient is unable to wear one, the question of some operative procedure arises. Of these he thinks the choice lies between ventrofixation, vaginal fixation, and shortening the round ligaments. J. Riddle Goffe[9] emphasises the importance of the utero-sacral, and the round ligaments, in maintaining the uterus in its normal position. He advocates the following operation for retrodisplacement. A transverse vaginal incision is made anterior to the vaginal portion of the cervix, and the peritoneum opened; through this opening the uterus is drawn, and first one round ligament and then the other shortened by looping it upon itself and stitching the folds together. J. W. Bovée[10] advocates shortening the utero-sacral ligaments for retroversion. He makes a transverse incision in the posterior vaginal fornix and exposes the ligaments. Each ligament is then folded upon itself and sutures passed to hold the folds in position, thus contracting the ligaments and drawing the cervix upwards and backwards. Some advocate attaching the ligaments to the fascia at the sides of the pelvis, but looping them together is generally thought to be preferable. H. N. Vineberg[11] considers that the operation of vaginal fixation still forms the ideal operation for certain cases of retroversion and retroflexion. The vaginal fixation, however, as a rule forms only one of a series of operations that are indicated, and which are performed at one sitting. The additional operations that are usually called for are (1) amputation of the cervix; (2) anterior colporrhaphy; (3) posterior colporrhaphy; (4) perineorrhaphy.

**Tubo-Ovarian Cysts.**—Preiser,[12] reviewing the different theories regarding the origin of these cysts, thinks that some are undoubtedly formed by the union of a pyosalpinx with either a follicular corpus luteum, or a proliferating cyst of the ovary. In another variety in which the fimbriæ do not disappear, but are found within the ovarian cyst, he thinks an abscess forms around the dilated end of the tube, the fimbriæ floating free in the sac, or being attached to its inner wall. An ovarian cyst may become adherent to this abscess, with the formation of a tubo-ovarian cyst by the disappearance of the wall of separation.

**The Vaginal Route for Operation upon the Uterus and Appendages.**—J. H. Branham[13] advocates operation by the vaginal in preference to the abdominal route in cases of pelvic inflammation. He records one hundred cases of his own (operated upon by this method) with but three deaths. Extensive pelvic inflammation associated with pus formation is rapidly walled off from the general peritoneal cavity, and it is most important that this wall of adhesions should not be broken through on account of the risk of setting up general peritonitis. He maintains that vaginal drainage in these cases is more effectual and safer than abdominal. Moreover, an opening through the vagina is attended with a minimum amount of shock and hæmorrhage, and is thus indicated in extreme cases associated with severe toxic symptoms. For cases of pyosalpinx complicated with intraperitoneal abscess, Noble[14] also recommends simple vaginal incision and drainage. He thinks that if the abdomen should be opened in this class of case under a mistaken diagnosis it would be better to make vaginal drainage at once, and close the abdominal wound, without any attempt at removal of the diseased appendages.

**Etiology of Hydrosalpinx.**—Meedernoort[15] discusses the question as to whether hydrosalpinx can develop without a previous catarrhal inflammation of the tube, and he reports a case which occurred at the menopause, in which careful microscopical investigation showed no evidence of inflammatory change. He thinks that since the tube normally contains a small quantity of serous fluid, which is increased during menstruation, it follows that the hyperæmia of the tubal mucous membrane accompanying any abnormal condition of the uterus, especially fibromata, would naturally result in profuse secretion. If in these cases the distal end becomes occluded, as the result of slight inflammation in the neighbourhood, the tube becomes gradually distended with fluid.

**Intraperitoneal Hæmorrhage Incident to Eclopic Gestation.**—C. J. Cullingworth[16] discusses the morbid anatomy of the two forms of intraperitoneal hæmorrhage which may occur in connection with ectopic gestation, viz., the diffuse intraperitoneal hæmorrhage, and the non-diffuse or encysted. With regard to the diffuse form, he points out that it is nearly always due to rupture of the gestation sac, and that this rupture most frequently occurs at the placental site. The chronic villi which at first completely surround the ovum, become specially developed at this part, and penetrate the tube even to its outer coat. The result of this ingrowing of the chronic villi is that the muscular fibres become separated, and the tube wall at that spot sensibly weakened.

The separation of the muscular fibres is still further increased by an infiltration of large nucleated cells. It is easy to understand that rupture of the gestation sac is especially apt to occur at the spot where the tube is thus weakened. With regard to the morbid anatomy of the non-diffused or encysted form, there is as a rule no rupture of the tube, but the blood escapes from the dilated ostium abdominale of the tube, the ovum having in many cases been previously converted into a "tubal mole." In other words, the ordinary result of tubal rupture is a diffuse and highly dangerous intraperitoneal hæmorrhage, whilst the usual result of bleeding from the mouth of the tube is the formation of a hæmatocele.

**Fibromata of the Ovary.**—Rootier[17] reports six cases in his own practice, in all of which he diagnosed a solid tumour, but was unable to determine whether it was of uterine or ovarian origin. Ascites was only noted in one case. This patient was 62 years of age, but the other five were under 36. The benign character of these growths is shown by the fact that they are unilateral. Menstruation may or may not be affected, both menorrhagia and amenorrhœa being observed. Pain is variable, and seems to be due to purely mechanical causes.

[8] The Clin. Jour., Sept. 3, 1902. [9] Med. News, July 12, 1902. [10] Med. Rec., June 14, 1902. [11] Med. Rec., Sept. 6, 1902. [12] Amer. Jour. of Med. Sci., Aug., 1902. [13] Med. Rec., Oct. 4, 1902. [14] Treatment, June, 1902. [15] Amer. Jour. of Med. Sci., Oct., 1902. [16] Lancet, Nov. 8, 1902. [17] Amer. Jour. of Med. Sci., Sept., 1902.

